# Flaws in design, analysis and interpretation of Pfizer's antifungal trials of voriconazole and uncritical subsequent quotations

**DOI:** 10.1186/1745-6215-7-3

**Published:** 2006-01-19

**Authors:** Karsten J Jørgensen, Helle Krogh Johansen, Peter C Gøtzsche

**Affiliations:** 1 The Nordic Cochrane Centre, Rigshospitalet, Copenhagen, Denmark; 2 Institute of Medical Microbiology and Immunology, University of Copenhagen, Denmark

## Abstract

We have previously described how a series of trials sponsored by Pfizer of its antifungal drug, fluconazole, in cancer patients with neutropenia handicapped the control drug, amphotericin B, by flaws in design and analysis. We describe similar problems in two pivotal trials of Pfizer's new antifungal agent, voriconazole, published in a prestigious journal. In a non-inferiority trial, voriconazole was significantly inferior to liposomal amphothericin B, but the authors concluded that voriconazole was a suitable alternative. The second trial used amphothericin B deoxycholate as comparator, but handicapped the drug by not requiring pre-medication to reduce infusion-related toxicity or substitution with electrolytes and fluid to reduce nephrotoxicity, although the planned duration of treatment was 84 days. Voriconazole was given for 77 days on average, but the comparator for only 10 days, which precludes a meaningful comparison.

In a random sample of 50 references to these trials, we found that the unwarranted conclusions were mostly uncritically propagated. It was particularly surprising that relevant criticism raised by the FDA related to the first trial was only quoted once, and that none of the articles noted the obvious flaws in the design of the second trial.

We suggest that editors ensure that the abstract reflects fairly on the remainder of the paper, and that journals do not impose any time limit for accepting letters that point out serious weaknesses in a study that have not been noted before.

## Introduction

The antifungal agent amphotericin B is a highly effective drug [1,2] that is often used as comparator in trials of new antifungal drugs. We have previously described how a series of trials sponsored by Pfizer of its antifungal drug, fluconazole, in cancer patients with neutropenia handicapped the control drug, amphotericin B, by flaws in design and analysis [3]. Amphotericin B is usually given intravenously [1,2], but most of the patients in Pfizer's trials were randomized to oral amphotericin B, which is poorly absorbed and not an established treatment.

Three of these trials were large and comprised 43% of the patients that were available for our meta-analysis of amphotericin B versus fluconazole [3]. In these trials, patients had been randomised to three arms, the third drug being nystatin, but the results for amphotericin B were combined with the results for nystatin. This is surprising, since this drug is recognized as ineffective in these circumstances, which we confirmed in a separate meta-analysis of trials with nystatin [3]. Despite repeated requests, neither the trial authors nor Pfizer provided us with separate data for each of the three arms in these studies.

### Flaws in trials of voriconazole

We now report problems with the design and analysis of Pfizer's trials on its new antifungal agent, voriconazole. We identified two eligible trials for our systematic review of voriconazole [4]. They were both large, sponsored by Pfizer, and published in *The New England Journal of Medicine *in 2002 [5,6]. As with our previous review [3], the first authors declined or were unable to answer our questions and referred to Pfizer, which in this case provided a response.

The first trial was a non-inferiority trial that compared voriconazole to liposomal amphothericin B as empirical treatment of fever of unknown origin in neutropenic cancer patients, using a composite endpoint with 5 separate outcomes [5]. Voriconazole was inferior to liposomal amphothericin B according to the authors' pre-specified criteria, and even *significantly *inferior according to the pre-specified analysis plan, which staff at the FDA pointed out in a subsequent letter to the journal [7]. More patients died in the voriconazole group and a claimed significant reduction in so-called "breakthrough" fungal infections in favour of voriconazole disappeared when infections arbitrarily excluded from analysis were included. The authors defined breakthrough fungal infections as those confirmed more than 24 hours post-enrolment. The reason for a 24-hour cut-point to exclude baseline fungal infections from analysis was not explained. We searched the references provided as justification for this cut-point in the article but could not find any relevant information. We have not seen such a cut-point in any of the more than 70 other trials of antifungal therapy we have reviewed [1-4,8], and in a later study of caspofungin, the same first author now used a 48-hour cut-point for the same outcome, in the same journal, and again without any justification or explanation [9].

We believe the use of arbitrary cut-points creates bias. When we included baseline infections that persisted despite treatment, we found 15 vs. 23 infections (P = 0.27) [4], whereas the trial report noted 8 versus 21 infections (P = 0.02) [5]. Our analysis is not only unbiased, it is also the clinically relevant one, as patients with baseline infections are part of the clinical reality when patients are treated on suspicion of a fungal infection.

The abstract was highly misleading. Despite the fact that voriconazole was clearly inferior to amphothericin B, it concluded that "Voriconazole is a suitable alternative to amphothericin B preparations" and referred to "breakthrough" fungal infections and a significant difference in nephrotoxicity, arbitrarily defined as a 1.5-fold increase of baseline s-creatinine values, which disappeared completely when the usual definition of a 2-fold increase was used [5]. Twenty-nine versus 32 patients had a 2-fold increase, whereas 43 versus 80 patients experienced a 1.5-fold increase (P < 0.001).

The second trial was flawed by design. It compared voriconazole to amphothericin B deoxycholate in the treatment of invasive Aspergillus infections [6]. The deoxycholate preparation was used without requirements for pre-medication, which is generally advised to reduce infusion-related toxicity, or substitution with electrolytes and fluid as advised to reduce nephrotoxicity, which can be practically abolished by this precaution [10,11]. These omissions are particularly disturbing considering that the planned duration of treatment was 84 days. Voriconazole was given for 77 days on average, but the comparator for only 10 days, which precludes a meaningful comparison of the two drugs.

### References to the flawed trials

Both trial reports have been extensively quoted. As of 23 November 2005, the paper by Walsh et al. had been quoted 192 times and the paper by Herbrecht et al. 344 times [12]. We selected randomly 50 articles of any type, e.g. research reports, reviews, editorials and letters, that quoted a trial report (25 for each report) to elucidate whether the quotations reflected uncritically the main conclusions of the trial reports. Two articles that were coincidentally selected for both groups were replaced by the next on the randomisation list (see final list in the Additional file 1). We also looked at conflicts of interest, defined as being author of one of the quoted trial reports; being a Pfizer employee; being a consultant, speaker or on the advisory board for Pfizer; or being author of an article with no declaration of conflicts of interest, but having declared a conflict of interest related to Pfizer in another of the included articles.

Two authors independently evaluated each article and differences were settled by discussion. We excluded 10 articles that quoted Walsh et al. and 7 articles quoting Herbrecht et al. as they did not refer to any of the main conclusions, but to minor issues, e.g. hepatotoxicity.

Five of the 15 included articles that quoted Walsh et al. accepted their main conclusion whereas 7 noted that non-inferiority had not been demonstrated. Seven articles mentioned the confidence interval by Walsh et al., whereas only one article referred to the confidence interval published by the FDA. Six articles noted that voriconazole led to fewer break-through infections whereas one noted that this was not a protocol-defined successful outcome and another mentioned problems with the multiplicity of outcomes without allowance for multiple testing as also noted by the FDA. Two articles claimed that voriconazole had less nephrotoxicity whereas one noted that the proportions that had a doubling in serum creatinine was similar. Only 3 articles had authors with a conflict of interest, and only one criticism was raised in these.

None of the 18 included articles that quoted Herbrecht et al. raised any criticism of the design of this trial and only one mentioned that concerns had been raised about "differing durations of therapy, as well as the possibility of excessive interruptions in the amphotericin arm", but the authors went on to say, in the next sentence, that: "the conclusion that voriconazole is superior to amphotericin B for treatment of invasive aspergillosis has been welcomed by most clinicians" [13]. Eleven articles had authors with a conflict of interest.

## Discussion

We believe the available evidence cannot support a recommendation to use voriconazole instead of amphothericin B in immune-suppressed cancer patients as amphothericin B given under optimal circumstances was significantly better than voriconazole.

The authors of both trial reports nevertheless drew positive conclusions about voriconazole. Such positive but unwarranted conclusions can be very useful for the sponsor's marketing department, in particular when published in a prestigious journal with a high impact factor. Journals that allow publication of misleading papers and conclusions of industry-sponsored trials also benefit. First, the income from reprints can be very large [14]. Second, drug companies may orchestrate a large series of ghost-written papers [15] that quote the favourable conclusions. This practice may boost the journal's impact factor further, adding to the journal's high reputation, but in reality watering down the impact factor as a measure of quality and reliability.

We found that the unwarranted conclusions were mostly uncritically propagated in subsequent articles. It was particularly surprising that the relevant criticism raised by the FDA was only quoted once, and that none of the articles that quoted the trial report by Herbrect et al. noted the obvious flaws in the design of this trial. We can only guess what the reasons are for this, but wish to point out that the number of authors with conflicts of interest we identified is likely to be a gross underestimate. This is supported by the fact that *The New England Journal of Medicine *during two years had been able to solicit and publish only one Drug Therapy article on a novel form of treatment after the journal had introduced a policy that authors of reviews and editorials would not have any financial interest in a company (or its competitor) that makes a product discussed in the article [16]. The journal has therefore changed the policy to "any *significant *financial interest".

We believe it is a problem for health care that references to flawed research can be so uncritical as we demonstrated and suggest further research in this area. Other problems with references are that they often predominantly select those previous articles that had the most favourable findings [17-19] or conclusions [19,20], or they exaggerate or distort findings [19].

## Conclusion

We agree with the former editor of the British Medical Journal that medical journals can serve as an extension of the marketing arm of the pharmaceutical industry [14]. This is difficult to avoid, even for prestigious journals with careful peer review. However, it should be relatively easy to ensure that the abstract reflects fairly on the remainder of the paper, which is currently often not the case [21,22]. Furthermore, journals should not have any time limit for accepting letters that point out serious weaknesses in a study that have not been noted before [23].

## Competing interests

The author(s) declare that they have no competing interests.

## Authors' contributions

HKJ and PCG conceived the project. The first draft was written by KJJ and revised by HKJ and PCG.

## Supplementary Material

Additional File 1 contains 25 randomly selected references to the trial by Walsh et al. and 25 randomly selected references to the trial by Herbrecht et al. Click here for file
