# Supplementary material for: Flaws in design, analysis and interpretation of Pfizer's antifungal trials of voriconazole and uncritical subsequent quotations
Source: Trials. 2006 Jan 19;7:3. doi: 10.1186/1745-6215-7-3 (PMC1399447; doi:10.1186/1745-6215-7-3)
Supplement: Additional File 1 — contains 25 randomly selected references to the trial by Walsh et al. and 25 randomly selected references to the trial by Herbrecht et al. [file 1745-6215-7-3-S1.doc]

**References to the study by Walsh et al.**

1. Agrawal AK, Sherman LK. Voriconazole-induced musical hallucinations. *Infection* 2004;32:293-5.

2. Alfandari S, Leroy O, De Botton S, Yakoub-Agha I, Durand-Joly I, Leroy-Cotteau A, Beaucaire G. Management of aspergillosis in immunocompromised patients. Recommendations of Lille University Hospital - 4th Version - November 2004. *Medecine et Maladies Infectieuses* 2005;35:121-34.

3. Baden LR. Prevention and therapy of fungal infections in bone marrow transplantation. *Leukemia* 2003;17:1038-41.

4. Baden LR, Rubin RH. Fever, neutropenia, and the second law of thermodynamics*. Ann Intern Med* 2002;137:123-4.

5. Bag R. Fungal pneumonias in transplant recipients. *Curr Opin Pulm Med* 2003;9:193-8.

6. Charbonneau P. Efficacy and tolerance of current anti-aspergillosis treatments. *Journal de Mycologie Medicale* 2002;12:15-17.

7. Florea NR, Kuti JL, Quintiliani R. Voriconazole - a novel azole antifungal. *Formulary* 2002;37:389-99.

8. Fournier S, Pavageau W, Feuillhade M, Deplus S, Zagdanski AM, Verola O, Dombret H, Molina JM. Use of voriconazole to successfully treat disseminated Trichosporon asahii infection in a patient with acute myeloid leukaemia. *Eur J Clin Microbiol Infect Dis* 2002;21:892-6.

9. Girmenia C, Moleti ML, Micozzi A Iori AP, Barberi W, Foa R, Martino P. Breakthrough Candida krusei fungemia during fluconazole prophylaxis followed by breakthrough zygomycosis during caspofungin therapy in a patient with severe aplastic anemia who underwent stem cell transplantation. *J Clin Microbiol* 2005;43:5395-6.

10. Gothard P, Rogers TR. Voriconazole for serious fungal infections. *Int J Clin Pract* 2004;58:74-80.

11. Graybill JR. Voriconazole for candidosis: an important addition? *Lancet* 2005;366:1413-4.

12. Hayes-Lattin B, Maziarz RT. Update in the epidemiology, prophylaxis, and treatment of fungal infections in patients with hematologic disorders. *Leuk Lymphoma* 2004;45:669-80.

13. Kim JE, Perkins SL, Harris GJ. Voriconazole treatment of fungal scleritis and epibulbar abscess resulting from scleral buckle infection. *Arch Ophthalmol* 2003;121:735-7.

14. Klastersky J. Antifungal therapy in patients with fever and neutropenia--more rational and less empirical? *N Engl J Med* 2004;351:1445-7.

15. Kullberg BJ, Oude Lashof AM. Epidemiology of opportunistic invasive mycoses. *Eur J Med Res* 2002;7:183-91.

16. Mallie M, Bastide JM, Blancard A, Bonnin A, Bretagne S, Cambon M, Chandenier J, Chauveau V, Couprie B, Datry A, Feuilhade M, Grillot R, Guiguen C, Lavarde V, Letscher V, Linas MD, Michel A, Morin O, Paugam A, Piens M A, Raberin H, Tissot E, Toubas D, Wade A. In vitro susceptibility testing of Candida and Aspergillus spp. to voriconazole and other antifungal agents using Etest: results of a French multicentre study. *Int J Antimicrob Agents* 2005;25:321-8.

17. Perfect JR, Marr KA, Walsh TJ, Greenberg RN, Dupont B, De La Torre-Cisneros J, Just-Nubling G, Schlamm HT, Lutsar I, Espinel-Ingroff A, Johnson E. Voriconazole treatment for less-common, emerging, or refractory fungal infections. *Clin Infect Dis* 2003;36:1122-31.

18. Ritter J. [Amphotericin B and its lipid formulations]. *Mycoses* 2002;45 Suppl 3:34-8.

19. Rodriguez CA, Lujan-Zilbermann J, Woodard P, Andreansky M, Adderson EE. Successful treatment of disseminated fusariosis. *Bone Marrow Transplant* 2003;31:411-2.

20. Segal BH, Bow EJ, Menichetti F. Fungal infections in nontransplant patients with hematologic malignancies. *Infect Dis Clin North Am* 2002;16:935-64.

21. Shetty A, Barnes RA. New antifungal agents. *Hosp Med* 2004;65:76-9.

22. Vishnubhotla P, Ibrahim RB, Abidi MH, Chandrasekar PH. Fever and eosinophilia associated with voriconazole. *Ann Pharmacother* 2004;38:900-1.

23. Walsh TJ, Karlsson MO, Driscoll T, Arguedas AG, Adamson P, Saez-Llorens X, Vora AJ, Arrieta AC, Blumer J, Lutsar I, Milligan P, Wood N. Pharmacokinetics and safety of intravenous voriconazole in children after single- or multiple-dose administration. *Antimicrob Agents Chemother* 2004;48:2166-72.

24. Wingard JR, Leather H. Hepatotoxicity associated with antifungal therapy after bone marrow transplantation. *Clin Infect Dis* 2005;41:308-10.

25. Wong-Beringer A, Kriengkauykiat J. Systemic antifungal therapy: new options, new challenges. *Pharmacotherapy* 2003;23:1441-62.

**Refences to the study by Herbrecht et al.**

1. Akan H. Management of invasive fungal infections in neutropenic patients. *Hematology* 2005;10:234-7.

2. Antachopoulos C, Roilides E. Cytokines and fungal infections. *Br J Haematol* 2005;129:583-96.

3. Baddley JW, Pappas PG. Antifungal combination therapy: clinical potential. *Drugs* 2005;65:1461-80.

1. Chandrasekar P. Riches usher dilemmas: antifungal therapy in invasive aspergillosis. *Biol Blood Marrow Transplant* 2005;1:77-84.

5. Cordonnier C. Fungal infections: current diagnosis and treatment. *Hematol J*  2004;5:59-62.

6. Denning DW, Kibbler CC, Barnes RA. British Society for Medical Mycology proposed standards of care for patients with invasive fungal infections. *Lancet Infect Dis* 2003;3:230-40.

7. Filler SG, Yeaman MR, Sheppard DC. Tumor necrosis factor inhibition and invasive fungal infections. *Clin Infect Dis* 2005;41:208-12.

8. Hope WW, Denning DW. Invasive aspergillosis: current and future challenges in diagnosis and therapy. *Clin Microbiol Infect* 2004;10:2-4.

9. Jantunen E, Anttila VJ, Ruutu T. Aspergillus infections in allogeneic stem cell transplant recipients: have we made any progress? *Bone Marrow Transplant* 2002;30:925-9.

10. Kojima R, Kami M, Nannya Y, Kusumi E, Sakai M, Tanaka Y, Kanda Y, Mori S, Chiba S, Miyakoshi S, Tajima K, Hirai H, Taniguchi S, Sakamaki H, Takaue Y. Incidence of invasive aspergillosis after allogeneic hematopoietic stem cell transplantation with a reduced-intensity regimen compared with transplantation with a conventional regimen. *Biol Blood Marrow Transplant* 2004;10:645-52.

11. Kruger WH, Russmann B, de Wit M, Kroger N, Renges H, Sobottka I, Zander AR. Haemopoietic cell transplantation of patients with a history of deep or invasive fungal infection during prophylaxis with liposomal amphotericin B. *Acta Haematol* 2005;113:104-8.

12. Marr KA. New approaches to invasive fungal infections. *Curr Opin Hematol* 2003;10:445-50.

1. Mattner F, Chaberny IF, Weissbrodt H, Fischer S, Gastmeier P, Haubitz B, Gottlieb J, Mattner L, Strueber M. [Surveillance of invasive mold infections in lung transplant recipients: effect of antimycotic prophylaxis with itraconazole and voriconazole]. *Mycoses* 2005;48:51-5.

14. Najvar LK, Graybill JR, Cacciapuoti AF. Challenges in designing animal studies to detect antagonism of polyene activity - Authors' reply. *Antimicrob Agents Chemother* 2004;48:3211-2.

15. Patterson TF. Early use of antifungal therapy in high-risk patients. *Curr Opin Infect Dis* 2002;15:561-3.

16. Perfect JR. Nuances of new anti-Aspergillus antifungals. *Med Mycol* 2005;43:271-6.

17. Powers JH. Considerations in clinical trials of combination antifungal therapy. *Clin Infect Dis* 2004;39:228-235.

18. Rubenstein M, Levy ML, Metry D. Voriconazole-induced retinoid-like photosensitivity in children. *Pediatr Dermatol* 2004;21:675-8.

19. Rubin ZA, Somani J. New options for the treatment of invasive fungal infections. *Semin Oncol* 2004;31:91-8.

20. Ruhnke M. Mucosal and systemic fungal infections in patients with AIDS: prophylaxis and treatment. *Drugs* 2004;64:1163-80.

21. Schwartz S, Thiel E. CNS-aspergillosis: are there new treatment options? *Mycoses* 2003;46:8-14.

22. Steinbach WJ. Antifungal agents in children. *Pediatr Clin North Am* 2005;52:895-915

23. Steinbach WJ, Benjamin DK Jr, Kontoyiannis DP, Perfect JR, Lutsar I, Marr KA, Lionakis MS, Torres HA, Jafri H, Walsh TJ. Infections due to Aspergillus terreus: a multicenter retrospective analysis of 83 cases. *Clin Infect Dis* 2004;39:192-8.

24. Stone RM. The difficult problem of acute myeloid leukemia in the older adult. *CA Cancer J Clin* 2002;52:363-71.

25. Zuccotti G, Strasfeld L, Weinstock DM. New agents for the prevention of opportunistic infections in haematopoietic stem cell transplant recipients. *Expert Opin Pharmacother* 2005;6:1669-79.
